# Spinal mechanisms underlying potentiation of hindpaw responses observed after transient hindpaw ischemia in mice

**DOI:** 10.1038/srep11191

**Published:** 2015-07-13

**Authors:** Tatsunori Watanabe, Mika Sasaki, Seiji Komagata, Hiroaki Tsukano, Ryuichi Hishida, Tatsuro Kohno, Hiroshi Baba, Katsuei Shibuki

**Affiliations:** 1 Department of Neurophysiology, Brain Research Institute, Niigata University,1-757 Asahi-machi, Chuo-ku, Niigata 951-8585, Japan; 2 Department of Anesthesiology, School of Medicine, Niigata University, 1-757 Asahi-machi, Chuo-ku, Niigata 951-8510, Japan

## Abstract

Transient ischemia produces postischemic tingling sensation. Ischemia also produces nerve conduction block that may modulate spinal neural circuits. In the present study, reduced mechanical thresholds for hindpaw-withdrawal reflex were found in mice after transient hindpaw ischemia, which was produced by a high pressure applied around the hindpaw for 30 min. The reduction in the threshold was blocked by spinal application of LY354740, a specific agonist of group II metabotropic glutamate receptors. Neural activities in the spinal cord and the primary somatosensory cortex (S1) were investigated using activity-dependent changes in endogenous fluorescence derived from mitochondrial flavoproteins. Ischemic treatment induced potentiation of the ipsilateral spinal and contralateral S1 responses to hindpaw stimulation. Both types of potentiation were blocked by spinal application of LY354740. The contralateral S1 responses, abolished by lesioning the ipsilateral dorsal column, reappeared after ischemic treatment, indicating that postischemic tingling sensation reflects a sensory modality shift from tactile sensation to nociception in the spinal cord. Changes in neural responses were investigated during ischemic treatment in the contralateral spinal cord and the ipsilateral S1. Potentiation already appeared during ischemic treatment for 30 min. The present findings suggest that the postischemic potentiation shares spinal mechanisms, at least in part, with neuropathic pain.

The mechanism of postischemic tingling sensation has been attributed to enhanced excitability of peripheral nerves during recovery from ischemic conduction block^1^,^2^. However, nerve conduction block is known to quickly induce plasticity in the central nervous system in experimental animals^3^ and humans^4^,^5^,^6^. Cessation of low frequency spontaneous firing of Aβ tactile afferents after partial denervation produces potentiation of neural responses in the primary somatosensory cortex (S1) elicited via the remaining nerves^7^. Reduction in the mechanical thresholds for paw-withdrawal reflex is observed at the same time^7^. This reduction in mechanical thresholds can be inhibited by spinal application of LY354740^7^, a specific antagonist of group II metabotropic glutamate receptors (mGluRs)^8^. If similar spinal mechanisms are also responsible for postischemic tingling sensation, this should be abolished by spinal application of LY354740. In the present study, we reproduced postischemic mechanical allodynia in mice, and tested roles of spinal mechanisms using this model.

Neuropathic pain is produced by nerve injury in animal models^9^,^10^,^11^, and the resulting pain is usually evaluated using behavioral tests more than 24 h after the injury to allow the recovery of animals from surgical injury. Therefore, the findings obtained from behavioral tests are affected by complex cascades of inflammation and gene expression during the recovery period^12^,^13^,^14^. However, postischemic mechanical allodynia is induced very quickly after transient hindpaw ischemia. Thus, if neural changes reflecting postischemic tingling sensation are found, these changes may be useful for investigating the neural mechanisms that trigger neuropathic pain. Activity-dependent flavoprotein fluorescence signals reflect aerobic energy metabolism in mitochondria^15^, and are useful for investigating fine neural activities and plasticity^16^,^17^,^18^. Flavoprotein signals are resistant to and spontaneously recover from photobleaching^19^, and quantitatively reflect neural activities^20^,^21^. Spinal activities elicited by peripheral stimulation have been visualized using flavoprotein fluorescence signals^22^. An initial phase of neuropathic pain after partial denervation is observed as long-lasting potentiation of flavoprotein fluorescence signals in S1^7^. Furthermore, a modality shift from tactile sensation to nociception can be visualized as reappearance of S1 responses to tactile stimulation in mice with ipsilateral dorsal column lesioning^7^. It is because the tactile sensation is mediated via the ipsilateral dorsal column to the contralateral S1^23^, while nociception is mediated via the contralateral spinothalamic tract^24^. In the present study, we confirmed that such a modality shift from tactile sensation to nociception was produced within 30 min after ischemic treatment.

## Results

### Reduced mechanical thresholds for paw-withdrawal reflex after ischemic treatment

**Figure 1:**
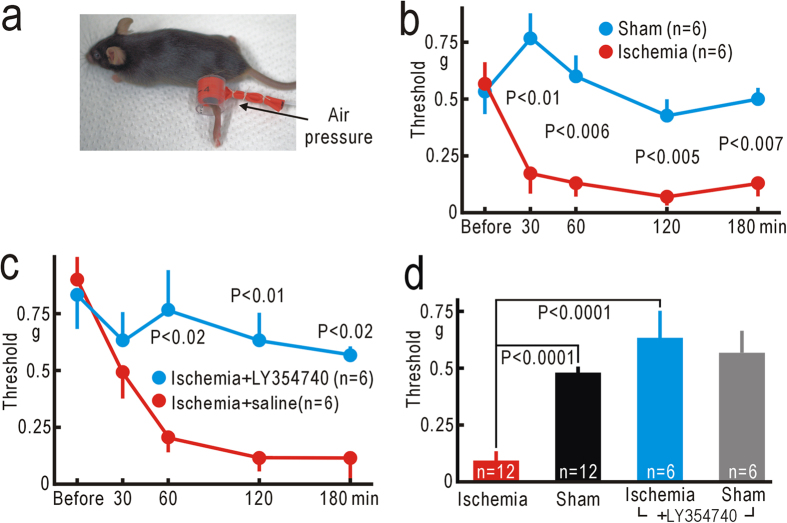
Reduced mechanical thresholds for hindpaw-withdrawal reflex. (**a**) Application of high pressure (250 mm Hg) to the left thigh. (**b**) Mechanical thresholds for left hindpaw-withdrawal reflex before and after ischemic or sham treatment applied to the left thigh. Mean ± SEM are shown. (**c**) Mechanical thresholds for hindpaw-withdrawal reflex after hindpaw ischemia in mice with spinal application of LY354740 (10 nM, 5 μl) or saline alone. (**d**) Comparison of the thresholds for the left hindpaw-withdrawal reflex at 2 h after ischemic or sham treatment, with or without spinal application of LY354740. In the ischemia group, data were mixed in mice with or without spinal application of saline. In the sham-treated group, data were mixed in mice with sham treatment to the left thigh or to the right thigh.

Because unpleasant postischemic tingling sensation is exacerbated by external mechanical forces, we measured the thresholds for hindpaw-withdrawal reflex using von Frey filaments^25^. The thresholds before hindpaw ischemia were 0.57 ± 0.10 g (mean ± SEM, n = 6). Mice were transiently anesthetized with 1% isoflurane, and a pressure of 250 mmHg was applied for 30 min to a rubber cuff set around the thigh (Fig. 1a). At 30 min after the ischemic treatment was finished, the mechanical thresholds were significantly reduced compared with the corresponding data in sham-treated mice with no pressure application (P < 0.01, Fig. 1b). The thresholds reached the minimal values of 0.07 ± 0.02 g (n = 6) at 2 h after the ischemic treatment (Fig. 1b), and were clearly and significantly smaller than the corresponding values in sham-treated mice (P < 0.005). Isoflurane anesthesia alone produced no reduction in the threshold in sham-treated mice (Fig. 1b).

Next, we tested whether the reduction in the thresholds was sensitive to spinal application of 10 nM LY354740 (Fig. 1c), which can block the initial phase of neuropathic pain after partial denervation^7^. When the ischemic treatment was applied to the mice that received spinal application of LY354740, the thresholds at 2 h after hindpaw ischemia were 0.63 ± 0.12 g (n = 6). In contrast, the thresholds were 0.12 ± 0.06 g (n = 6) in mice that received spinal application of saline alone (Fig. 1c). The difference at 2 h after hindpaw ischemia or sham-treatment was statistically significant (P < 0.01). We confirmed that spinal application of LY354740 alone had no apparent effect on the mechanical thresholds for paw-withdrawal reflex (Fig. 1d). Interestingly, a slight but significant reduction in the threshold was observed in the right hindpaw (Supplementary Fig. S1), suggesting that transient hindpaw ischemia might also have some effects on the contralateral spinal cord.

### Flavoprotein fluorescence responses in the spinal cord

**Figure 2:**
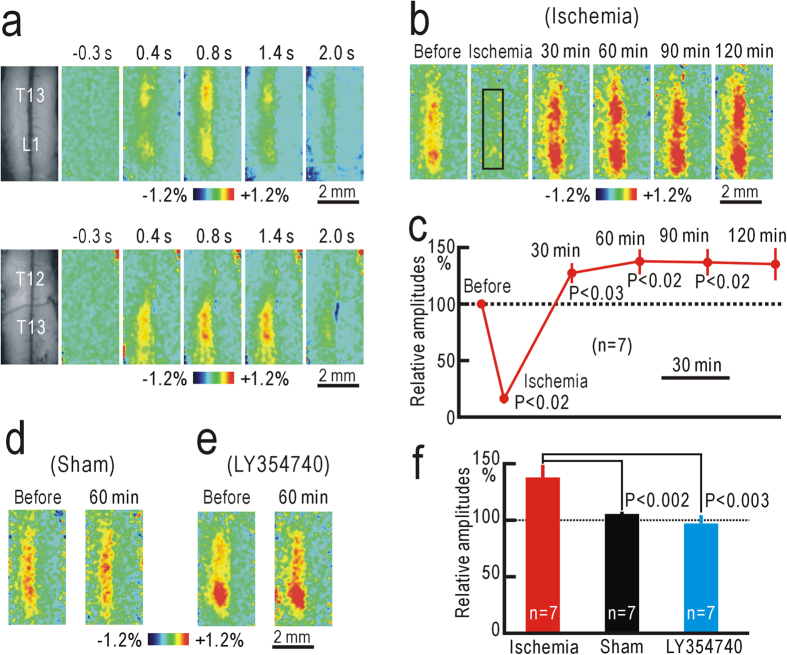
Potentiation of the spinal responses after hindpaw ischemia. (**a**) Example of ipsilateral spinal responses elicited by vibratory stimulation applied to the left hindpaw at T13 and L1 level (upper panels). The left-most panels are original fluorescence images, and others are pseudocolor images of response magnitudes in ΔF/F_0_ recorded at the time before and after the stimulus onset shown on each image. Another example of ipsilateral spinal responses elicited by vibratory stimulation applied to the left hindpaw at T12 and T13 level (lower panels). (**b**) Example of spinal responses recorded in the same mouse before, during, and 30–120 min after ischemic treatment applied to the left thigh for 30 min. The response amplitudes were measured in the square window of 100 × 25 pixels shown in the second panel. (**c**) Relative amplitudes of spinal responses during and after ischemic treatment. The amplitudes were normalized by those recorded before hindpaw ischemia. (**d**) Example of spinal responses recorded before and at 60 min after sham treatment. (**e**) Example of spinal responses recorded before and at 60 min after hindpaw ischemia in a mouse with spinal application of 10 nM LY354740. (**f**) Comparison of the normalized response amplitudes in mice at 60 min after hindpaw ischemia, sham treatment, or hindpaw ischemia with spinal application of 10 nM LY354740.

Because the behavioral test data suggested the presence of some spinal plasticity, we recorded ipsilateral spinal responses elicited by vibratory hindpaw stimulation using flavoprotein fluorescence imaging. Hindpaw stimulation produced an increase in fluorescence on the dorsal surface of the ipsilateral spinal cord corresponding to the dorsal horn at the T13 and L1 level (Fig. 2a). Because minimal response was found at T12, the fluorescence changes were attributed mainly to localized activities in the dorsal horn but not to ascending afferent activities mediated via the dorsal column. The responses started at 0.2 s after the onset of the stimulation, and peaked at approximately 1% in ΔF/F_0_ at 0.6–0.8 s after the stimulus onset. Although these properties were similar to those recorded in cortical areas^7^,^26^, the decay was slower than that of the cortical responses, as the hemodynamic responses were not marked in the spinal cord (Supplementary Figs S2).

The spinal responses to vibratory hindpaw stimulation were abolished by a pressure of 250 mmHg applied to the thigh, indicating that ischemic conduction block was successfully produced at 250 mmHg (Fig. 2b). The responses reappeared within 30 min after hindpaw ischemia, and the magnitude of the responses estimated at 30 min after hindpaw ischemia was slightly but significantly potentiated (P < 0.03, Fig. 2b,c). At 1 h after hindpaw ischemia, the response amplitudes reached maximal values, and were potentiated to 138 ± 11% (n = 7) compared with those before hindpaw ischemia (Fig. 2c). No clear potentiation was observed in sham-treated mice, and the difference between the two groups was statistically significant (P < 0.002, Fig. 2d,f).

Next, we applied 10 nM LY354740 on the surface of the spinal cord during imaging experiments. Application of LY354740 had no immediate effect on the spinal responses to vibratory hindpaw stimulation (Fig. 2e). However, the spinal responses were not clearly potentiated after hindpaw ischemia, and the relative amplitudes at 1 h after hindpaw ischemia were significantly smaller than those in mice with no LY354740 (P < 0.003, Fig. 2e,f). These data suggest that transient hindpaw ischemia produced spinal potentiation, and the reduction in mechanical thresholds for paw-withdrawal reflex, likely via similar spinal mechanisms sensitive to LY354740.

The spinal responses occasionally appeared in separated segments (for example, Fig. 2a), which might reflect the presence of functional units. Therefore, we tested the fine somatotopic maps in the spinal responses elicited by vibratory stimulation applied to each toe. The spinal responses to stimulation of each toe appeared in small areas that were different each other (Supplementary Fig. S2). The responsive areas were arranged almost linearly (Supplementary Figs S2). These results were compatible with a previous morphological study on spinal distribution of afferent fibers from each digit^27^. Therefore, the apparent segments in the spinal responses likely reflected strong stimulation of the skin convexes by the brush used for hindpaw stimulation.

### Flavoprotein fluorescence responses in S1

**Figure 3:**
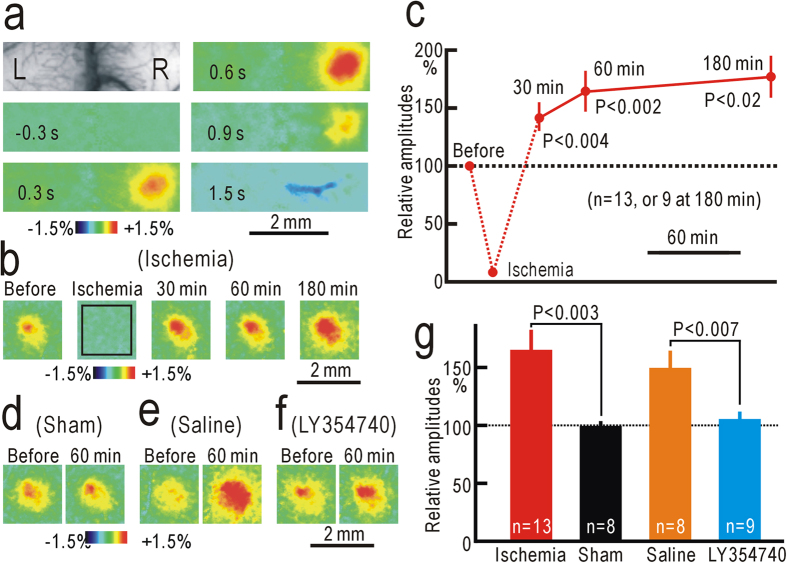
Potentiation of the S1 responses after ischemic treatment. (**a**) Example of contralateral S1 responses elicited by vibratory stimulation applied to the left hindpaw. (**b**) Example of S1 responses recorded in the same mouse before, during, and 30–180 min after ischemia applied to the left thigh for 30 min. The response amplitudes were measured in the square window of 60 × 60 pixels shown in the second panel. (**c**) Relative amplitudes of S1 responses during and after hindpaw ischemia. (**d**) Example of S1 responses recorded before and at 60 min after sham treatment. (**e**) Example of S1 responses recorded before and at 60 min after hindpaw ischemia in a mouse with spinal application of saline alone. (**f**) Example of S1 responses recorded before and at 60 min after hindpaw ischemia in a mouse with spinal application of 10 nM LY354740. (**g**) Comparison of the normalized response amplitudes in mice at 60 min after hindpaw ischemia, sham treatment, hindpaw ischemia with spinal application of saline alone, or hindpaw ischemia with spinal application of 10 nM LY354740.

Flavoprotein fluorescence responses elicited by hindpaw stimulation appeared in the contralateral S1 (Fig. 3a). The initial time course of S1 responses elicited by hindpaw stimulation was similar to that of spinal responses, while hemodynamic responses obscured the later phase of the responses (Supplementary Figs S3). Although neural responses in S1 are arranged according to somatotopic maps over a large scale^26^,^28^,^29^, the responses elicited by stimulation of each toe were not clearly separated, and no fine somatotopic map was found (Supplementary Figs S3). When a pressure of 250 mmHg was applied to the thigh, S1 responses were almost completely abolished (Fig. 3b). However, the responses reappeared and were potentiated after hindpaw ischemia (Fig. 3b,c). The potentiation was maintained up to 3 h after hindpaw ischemia. The relative amplitudes at 1 h after hindpaw ischemia (165 ± 18%, n = 13) were significantly larger than those in sham-treated mice (99 ± 4%, n = 8, P < 0.003, Fig. 3d,g). Potentiation of S1 responses after ischemic treatment was almost completely abolished by spinal application of 10 nM LY354740 (Fig. 3e,f). The relative amplitudes of S1 responses in mice with spinal application with LY354740 were 105 ± 6% (n = 9) at 1 h after hindpaw ischemia, and were significantly smaller than those in mice with spinal application of saline alone (150 ± 16%, n = 8, P < 0.007, Fig. 3g). Therefore, the suppression of S1 potentiation is likely attributable to the pharmacological effects of LY354740, but not to non-specific spinal injury caused by the drug application.

### Modality shift from tactile sensation to nociception induced by hindpaw ischemia

**Figure 4:**
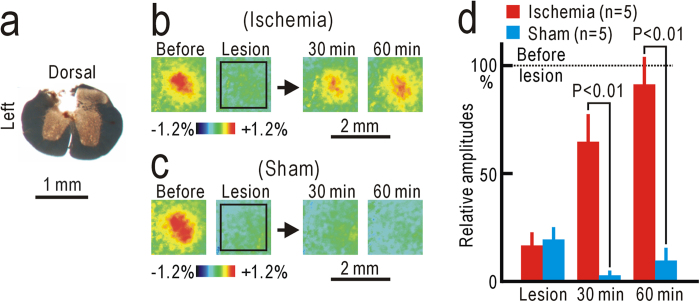
Sensory modality shift induced by ischemic treatment. (**a**) Lesion in the left dorsal column at T11 level. (**b**) Example of the right S1 responses modified by the left column lesioning and ischemic treatment applied to the left thigh. (**c**) Example of the right S1 responses modified by the left column lessoning and sham treatment applied to the left thigh. (**d**) Response amplitudes immediately after dorsal column lesioning, and at 30 min and 60 min after ischemic or sham treatment.

Potentiation in S1 after ischemic treatment may be produced by a modality shift from tactile sensation to nociception, as observed after partial denervation^7^. When we disrupted the ipsilateral dorsal column at the T11 level (Fig. 4a), S1 responses elicited by hindpaw stimulation were abolished (Fig. 4b). However, S1 responses reappeared at 30 and 60 min after ischemic treatment applied to the hindpaw (Fig. 4b). As no such response was observed in sham-treated mice (Fig. 4c), the reappearing S1 responses cannot be attributed to spontaneous recovery from the injury caused by dorsal column lesioning. The difference in S1 responses in the operated mice was statistically significant at 30 and 60 min after hindpaw ischemia or sham treatment (P < 0.01 for both, Fig. 4d). The reappearing S1 responses suggest that the modality shift from tactile sensation to nociception was induced within 30 min after ischemic treatment.

### Potentiation in the contralateral spinal cord and the ipsilateral S1 during hindpaw ischemia

**Figure 5:**
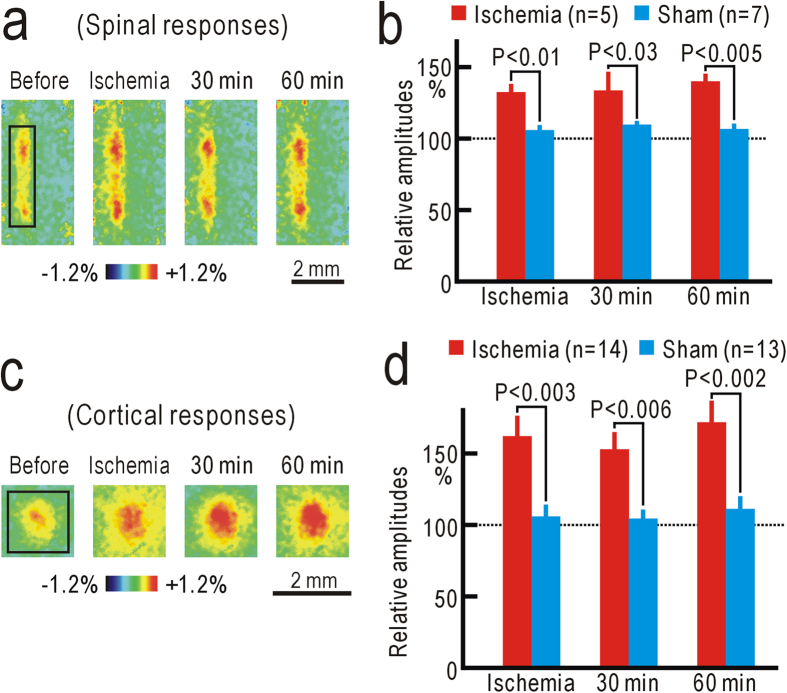
Responses to peripheral stimulation contralateral to hindpaw ischemia. (**a**) Example of spinal responses to left hindpaw stimulation before, during, and at 30 min and 60 min after ischemic treatment applied to the right hindpaw. (**b**) Relative amplitudes of the responses to left hindpaw stimulation before, during, and at 30 min and 60 min after ischemic or sham treatment applied to the right hindpaw. Responses were normalized by those recorded before ischemic or sham treatment. (**c**) Example of S1 responses to left hindpaw stimulation before, during, and after ischemic treatment applied to the right hindpaw. (**d**) Relative amplitudes of the responses to left hindpaw stimulation.

Changes in neural responses were not observable during ischemic treatment in the ipsilateral spinal cord or in the contralateral S1 because of conduction block of the peripheral nerves. However, we found that the spinal responses elicited by vibratory stimulation applied to the hindpaw contralateral to the ischemic treatment were significantly potentiated during ischemic treatment (Fig. 5a,b). The potentiation was maintained for at least 60 min after hindpaw ischemia. Cortical responses in S1 ipsilateral to the ischemic treatment were similarly potentiated during and after hindpaw ischemia (Fig. 5c,d), indicating that spinal and cortical potentiation had already been initiated during ischemic treatment. Comparison of the potentiation at 60 min after hindpaw ischemia in the spinal cord (ipsilateral: 138 ± 11%, n = 7; contralateral: 140 ± 6%, n = 5) and S1 (contralateral: 165 ± 18%, n = 13; ipsilateral; 171 ± 14%, n = 14) revealed that the neural changes in the contralateral spinal cord and the ipsilateral S1 were comparable to those in the opposite sides. These results suggest that reduction in the mechanical thresholds of the right hindpaw-withdrawal reflex could be observed after ischemic treatment applied to the left thigh. In accordance with these results, a slight but significant reduction in the mechanical threshold (P < 0.02) was observed in the right hindpaw withdrawal reflex at 3 h after ischemia applied to the left hindpaw. However, S1 responses to forepaw stimulation were not clearly affected by ischemia applied to the hindpaw (Supplementary Figs S4).

## Discussion

### Postischemic mechanical allodynia produced by spinal mechanisms in mice

In the present study, we found that mechanical thresholds for hindpaw-withdrawal reflex were reduced after ischemic treatment. Previous studies also reported that hyperalgesia^30^ or mechanical allodynia^31^ is observed after hindpaw ischemia in animal experiments. However, it is difficult to differentiate between tingling sensation (or dysesthesia) and hyperalgesia or mechanical allodynia using behavioral tests, because all of these symptoms produce reduced mechanical thresholds. The reduced mechanical thresholds after ischemic treatment in the present study can be regarded as dysesthesia, because tingling sensation rather than pain is frequently experienced by human subjects after reversible hindpaw ischemia. Usually, postischemic dysesthesia due to hyperexcitability of peripheral nerves continues for approximately 5 min after ischemic treatment^1^, while use of a tourniquet to prevent bleeding for more than 2 h can produce long-lasting dysesthesia^32^.

The reduced mechanical thresholds were accompanied by potentiation in the spinal cord and S1. These three changes after ischemic treatment were abolished by spinal application of LY354740. Spinal and S1 responses to the right hindpaw stimulation were also potentiated during ischemia applied to the left hindpaw. Although long lasting-ischemia and reperfusion produce peroxides and resulting cytokine-mediated adverse pathophysiological effects on peripheral tissues^33^,^34^, potentiation in the contralateral spinal cord or in the ipsilateral S1 was observed before reperfusion was started, and S1 responses to forepaw stimulation were not affected by ischemia applied to the hindpaw. Taken together, these data suggest that postischemic tingling sensation was produced by localized spinal mechanisms in our mouse model.

### Spinal neural circuits and mechanisms producing postischemic plasticity

Neural imaging is useful for investigating mechanisms underlying postischemic tingling sensation. We used endogenous fluorescence signals derived from the mitochondrial flavoproteins^15^. Because flavoproteins are endogenous proteins of the electron transfer system in the mitochondria, it is very unlikely that flavoproteins work as a calcium chelator and have some artificial effects on the calcium dynamics, which play essential roles in induction of synaptic plasticity. We have successfully recorded various types of neural plasticity using this imaging method^16^,^17^,^20^,^35^. Flavoprotein fluorescence responses are mainly derived from synaptically-driven neuronal activity^21^, and no clear fluorescence response was found from the ipsilateral dorsal column that mediates tactile sensory information during hindpaw stimulation. Therefore, the spinal flavoprotein fluorescence responses were presumably derived from dorsal parts of the dorsal horn, which is composed of lamina I–VI^36^. Of these, tactile inputs mediated by Aβ fibers are terminated mainly in lamina III–V. Lamina II, or the substantial gelatinosa, has a critical role in neuropathic pain^37^,^38^, and receives nociceptive inputs mediated mainly by C fibers^39^. Group II mGluRs are located in the substantial gelatinosa^40^. Taken together, the spinal fluorescence responses elicited by tactile stimuli to the hindpaw are likely derived from neuronal activity in lamina III–V, and the potentiation observed after ischemic treatment likely reflects the recruitment of neuronal activity in the substantial gelatinosa.

**Figure 6:**
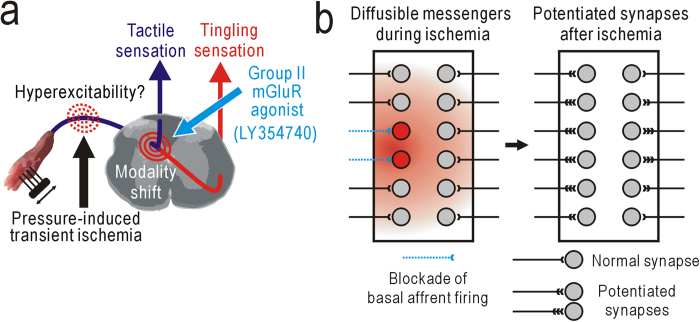
Mechanisms underlying postischemic plasticity. (**a**) Sensory modality shift from tactile to tingling sensation at the spinal cord level. (**b**) Schematic drawings of spinal potentiation ipsilateral and contralateral to ischemic treatment. Expected spinal potentiation induced after partial nerve cutting may also be induced by similar mechanisms.

An initial phase of neuropathic pain after partial denervation is abolished by spinal application of LY354740^7^, and this compound and similar group II mGluR agonists alleviate neuropathic pain^8^,^41^. Because the potentiation in the present study was also susceptible to spinal application of LY354740, these two types of potentiation may share similar mechanisms, at least in part, with neuropathic pain (Fig. 6a). Activation of postsynaptic group II mGluRs reduces presynaptic transmitter release^42^, produces membrane hyperpolarization by opening inward rectifier K^+^ channels^43^,^44^, and modulates spontaneous Ca^2+^ spikes^45^. Therefore, failure of group II mGluR activation during conduction block of peripheral nerve activities may increase the excitability and intracellular Ca^2+^ concentration of postsynaptic neurons. These changes induce down-regulation of the neuron-specific KCl cotransporter (KCC2) in dorsal horn neurons and a resulting reduced neuronal Cl^–^ gradient^46^,^47^,^48^. Nociceptive spinal neurons are dynamically regulated by inhibition from various sources^49^,^50^, and modulation of the Cl^–^ gradient can explain the increased responsiveness of spinothalamic tract neurons to innocuous mechanical stimuli in animals with neuropathic pain^48^,^51^. The spinal potentiation is induced not only in the ipsilateral site that fails to receive basal afferent firing, but also in the contralateral site or nearby site after partial nerve cutting. Because stimulation of different skin areas produces separate spinal responses (for example, Fig. 3), the potentiation in nearby spinal sites may have been induced by diffusible mediators produced in spinal neurons that failed to receive basal afferent firing (Fig. 6b). It is suggested that hindpaw ischemia produced some diffusible mediators that produced not only potentiation of contralateral hindpaw responses but also contralateral mechanical allodynia. Diffusible mediators may also play a role in potentiation of spinal responses ipsilateral to ischemic treatment. In accordance with this concept, neuropathic pain was reported to be facilitated by a number of diffusible mediators such as nitric oxide^52^ and ATP/cytokines^53^. The mechanical allodynia observed after hindpaw ischemia seemed to be more clearly observed in mice compared with similar phenomena in humans. Furthermore, humans do not clearly exhibit tingling sensation contralateral to the ischemic side or after partial nerve cutting. This difference may be attributed to the small size of spinal circuits in mice, in which diffusible messengers such as nitric oxide can be very effective. Another important consideration is that the potentiation observed during ischemic treatment in the present study could not be induced by repetitive activity in peripheral nerves, another condition known to induce spinal potentiation^54^,^55^.

### Postischemic changes as an experimental model for investigating neuropathic pain

Neuropathic pain is usually induced by peripheral nerve injury in animal models^9^,^10^,^11^. It is produced by a complex cascade of inflammation and gene expression, and not only neurons but also glial cells in the spinal cord have important roles in neuropathic pain^12^,^13^,^14^. We have reported that an initial phase of neuropathic pain is observed at a few hours after partial denervation^7^. However, postischemic tingling sensation and plasticity, which share similar spinal mechanisms with neuropathic pain, were observed at 30 min after reversible hindpaw ischemia. Furthermore, potentiation of spinal responses contralateral to the ischemic side appeared within 30 min of ischemic treatment. Regardless of the species difference, the very early onset of neural plasticity during reversible hindpaw ischemia or transient functional deafferentation in mice make them useful as an experimental model for observing and investigating the detailed cellular and molecular cascades that trigger human neuropathic pain. However, further studies on neuronal and glial activities in the spinal cord are required for establishing a new preclinical model of early neuropathic pain based on the present studies.

## Materials and methods

The experiments in the present study were approved by the ethics committee of animal experiments in Niigata University (approved number: 233-4), and were carried out in accordance with the approved guidelines. Male C57BL/6 mice between 7 and 10 weeks old, purchased from Charles River Japan (Tokyo, Japan), were used in the present study.

### Estimation of mechanical thresholds for hindpaw-withdrawal reflex

The mechanical thresholds for hindpaw-withdrawal reflex were measured using von Frey filaments^25^. The forces produced by von Frey filaments were between 0.008 and 1.4 g (respective sizes: between 1.65 and 4.17). Mice were separately placed into a transparent plastic box with a mesh floor, and accustomed to the state for 30 min. The thresholds were determined from the minimal force at which hindpaw withdrawal reflex was induced more than twice in eight trials. Transient ischemia was applied to the left hindpaw of mice lightly anesthetized with 1% isoflurane. Urethane anesthesia (1.65 g/kg, i.p.) was also used for imaging experiments. A small rubber cuff was set around the left thigh, and covered with a hard plastic tube (Fig. 1a). Air pressure at 250 mmHg was applied for 30 min to the tubing connected to the cuff using a mercury manometer. The pressure was directed to the thigh, because inflation of the rubber cuff was limited by the hard plastic tube. We confirmed that this treatment was sufficient to produce transient ischemia of the hindpaw, as neural responses elicited by vibratory stimulation to the hindpaw were blocked by this manipulation (for example, Figs 2c,4b). No apparent impairment except reduced threshold of paw-withdrawal reflex was found in the left leg after the mice recovered from isoflurane anesthesia.

### Imaging experiments

The surgical procedures were performed as described previously^7^. Mice were anesthetized with urethane (1.65 g/kg, i.p.), and a tracheotomy was performed for facilitating spontaneous respiration. During the experiments, body temperature was monitored using a rectal probe and maintained at 38 °C using a silicon rubber heater. These surgical operations were usually finished within 60 min. Recordings were started at 30 min after the surgical operations. Additional doses of urethane (0.1–0.2 g/kg, s.c.) were administered when necessary. When spinal responses to hindpaw stimulation were investigated, the vertebral arch was removed at the T13 and L1 level, and the dorsal surface of the spinal cord with the intact dura mater was exposed. The surface was cleaned with saline, and covered with 2% agarose to prevent spinal movement. The surface of the agarose gel was covered with a mixture of petroleum jelly and liquid paraffin to prevent drying. The spinal cord was fixed under a microscope using a clamp (STS-A; Narishige, Tokyo, Japan). Spontaneous respiration was maintained during the imaging experiment, because the movement of the spinal cord caused by respiration was minimal. Flavoprotein fluorescence imaging was performed as described previously^7^. Endogenous green fluorescence (λ = 500–550 nm) was recorded in blue light (λ = 450–490 nm). Images (128 × 168 pixels) of the spinal cord or S1 were recorded at 9 frames/s using a cooled charge coupled device camera (ORCA-R2; Hamamatsu Photonics, Hamamatsu, Japan). The camera was attached to a binocular epifluorescence microscope (M165 FC; Leica Microsystems, Wetzlar, Germany) with a 75-W xenon light source and a 1× objective lens. Serial images were taken in recording sessions repeated at 50 s intervals. Brush vibration (amplitude: 0.2 mm; frequency: 50 Hz) was applied for 600 ms to the sole of the hindpaw using a mechanical stimulator (DPS-290; Dia Medical, Tokyo, Japan). When fine somatotopic maps were investigated in the spinal cord or S1, brush vibration was applied to each toe. Fluorescence changes elicited by the stimulation were averaged over 24 trials. Because it took approximately 20 min to obtain data from 24 trials, the recording time of the averaged data was defined as the middle point of the recording period. Spatial averaging in 5 × 5 pixels and temporal averaging in three consecutive frames were used for smoothing and improving the image quality. The images were normalized, pixel by pixel, with respect to a reference image, which was obtained by averaging five images taken immediately before the stimulation. In the figures, parts of the normalized images are shown in a pseudocolor scale representing the fractional fluorescence changes (ΔF/F_0_). The response amplitude was evaluated as values of ΔF/F_0_ in a square window of 100 × 25 pixels or 3.84 × 0.96 mm. The location of the window was adjusted to maximize the response amplitude in ΔF/F_0_. After the recordings, mice were euthanized with an overdose of pentobarbital (300 mg/kg, i.p.).

For investigating the S1 responses to hindpaw stimulation, the disinfected head skin was incised, and the skull over the right S1 was exposed. The surface of the skull was cleaned with sterile saline, and a small piece of metal was attached to the skull with dental acrylic resin (Super Bond; Sun Medical, Shiga, Japan) to fix the head under a microscope. The surface of the skull was covered with a mixture of petroleum jelly and liquid paraffin to keep the skull transparent. The response amplitude was evaluated as values of ΔF/F_0_ in a square window of 60 × 60 pixels or 1.55 × 1.55 mm.

### Dorsal column lesioning

The left dorsal column was disrupted using an ultrasonic cutter (NE87; NSK, Kanuma, Japan) at the T11 level, as described previously^7^. To verify the lesion, the spinal cord was isolated after the recording experiments and fixed with 10% paraformaldehyde. Serial spinal cord sections of 100-μm thickness were cut using a microslicer (PRO-7; Dosaka, Kyoto, Japan), and the translucent images were observed.

### Spinal application of LY354740 to the spinal cord

In behavioral experiments, LY354740 (10 nM, 5 μl), obtained from Santa Cruz Biotechnology (Santa Cruz, USA), was applied to the spinal cord with intrathecal injection before hindpaw ischemia. In imaging experiments of the spinal responses, epidural application and infiltration of 10 nM LY354740 for at least 30 min was performed before the epidural surface was covered with 2% agarose. Epidural application and infiltration of saline alone was naturally performed in other imaging experiments of the spinal responses. In imaging experiments of S1 responses, the epidural application and infiltration of 10 nM LY354740 or saline alone was performed at the T13–L1 level.

### Statistics

Statistical significance in data was analyzed using StatView software (SAS Institute Inc., Cary, USA). Unpaired data obtained from different mice were evaluated by the Mann Whitney U-test. Paired data obtained from the same mice were evaluated by the Wilcoxon signed rank test. Only P values less than 0.05 are shown.

## Additional Information

**How to cite this article**: Watanabe, T. *et al.* Spinal mechanisms underlying potentiation of hindpaw responses observed after transient hindpaw ischemia in mice. *Sci. Rep.* **5**, 11191; doi: 10.1038/srep11191 (2015).

## Supplementary Material

Supplementary Information
