# Supplementary material for: Spinal mechanisms underlying potentiation of hindpaw responses observed after transient hindpaw ischemia in mice
Source: Sci Rep. 2015 Jul 13;5:11191. doi: 10.1038/srep11191 (PMC4499883; doi:10.1038/srep11191)
Supplement: Supplementary Information [file srep11191-s1.pdf]

Supplementary Figures S1 – S4

Spinal mechanisms underlying potentiation of hindpaw responses observed after transient hindpaw ischemia in mice

Tatsunori Watanabe[1,2], Mika Sasaki[2], Seiji Komagata[1], Hiroaki Tsukano[1], Ryuichi Hishida[1], Tatsuro Kohno[2], Hiroshi Baba[2], Katsuei Shibuki[1]

1. Department of Neurophysiology, Brain Research Institute, Niigata University, 1-757 Asahi-machi, Chuo-ku, Niigata 951-8585, Japan
2. Department of Anesthesiology, School of Medicine, Niigata University, 1-757 Asahi-machi, Chuo-ku, Niigata 951-8510, Japan

Correspondence: Katsuei Shibuki

Department of Neurophysiology, Brain Research Institute, Niigata University

1-757 Asahi-machi, Chuo-ku, Niigata 951-8585, Japan

E-mail: shibuki@bri.niigata-u.ac.jp; Phone: +81-25-227-0625; Fax: +81-25-227-0628

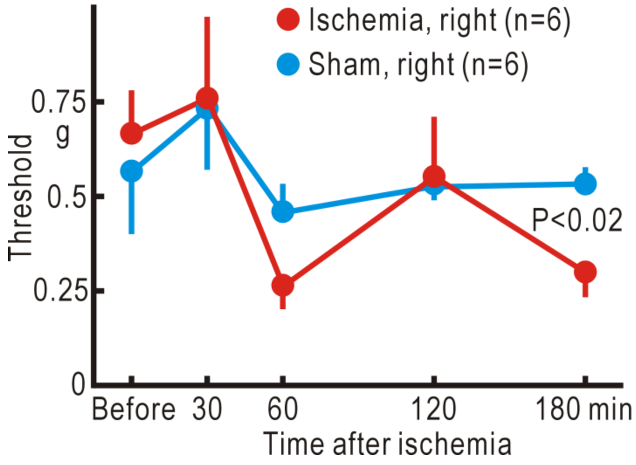

Supplementary Figure S1. Reduced mechanical thresholds for hindpaw-withdrawal reflex contralateral to ischemic treatment. Mechanical thresholds for left hindpaw-withdrawal reflex before and after ischemic or sham treatment applied to the right thigh. A significant difference (P<0.02) was observed at 3 h after the treatments.

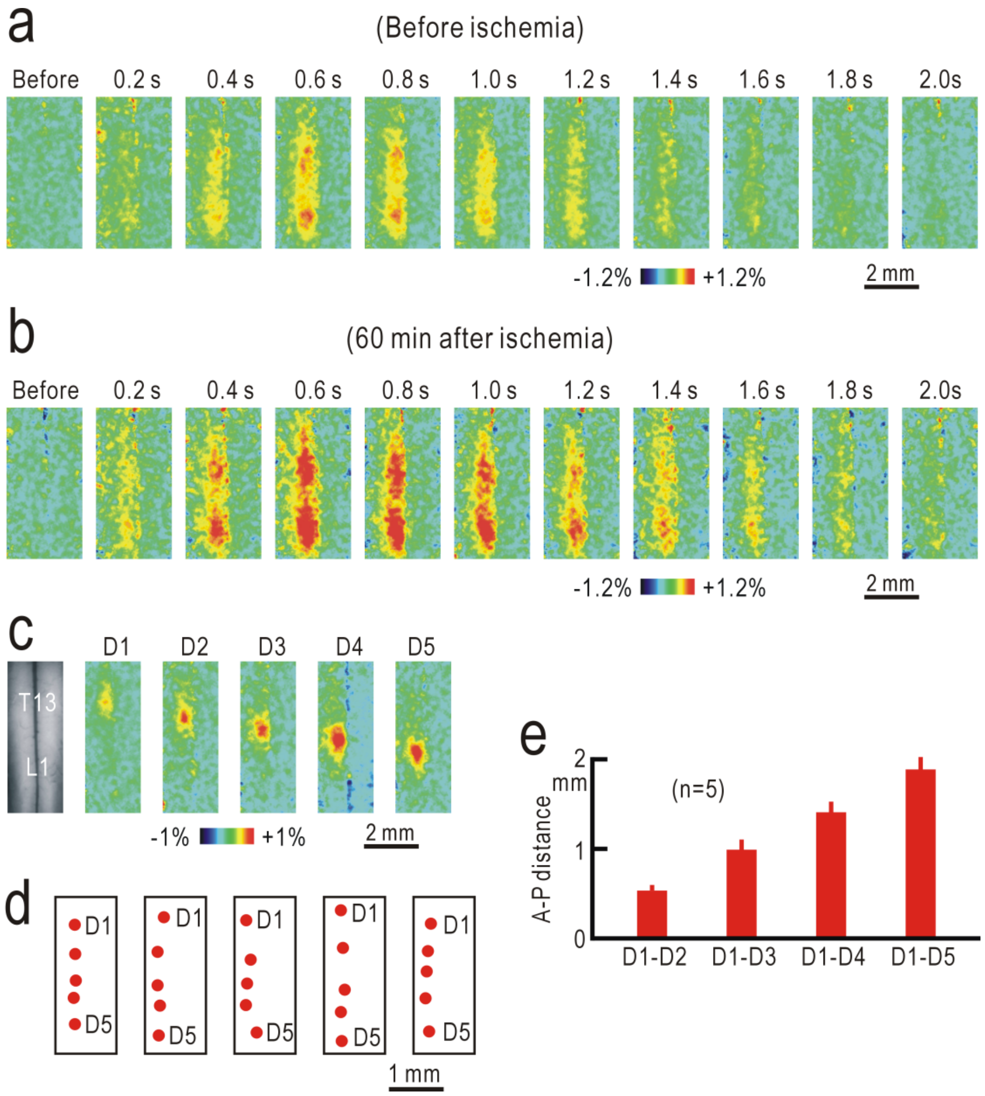

Supplementary Figure S2. Properties of spinal responses. (a) Sequential spinal images shown at 0.2 s intervals before ischemic treatment. **(b)** Sequential images shown at 0.2 s intervals 60 min after ischemic treatment. These images were obtained from the same mouse shown in Fig. 2b. **(c)** Example of ipsilateral spinal responses elicited by stimulation of each toe in the left hindpaw. **(d)** Somatotopic maps obtained from five different mice. The locations of the response center were plotted. **(e)** Anterior-posterior (A-P) distance between the locations of the spinal responses elicited by each toe.

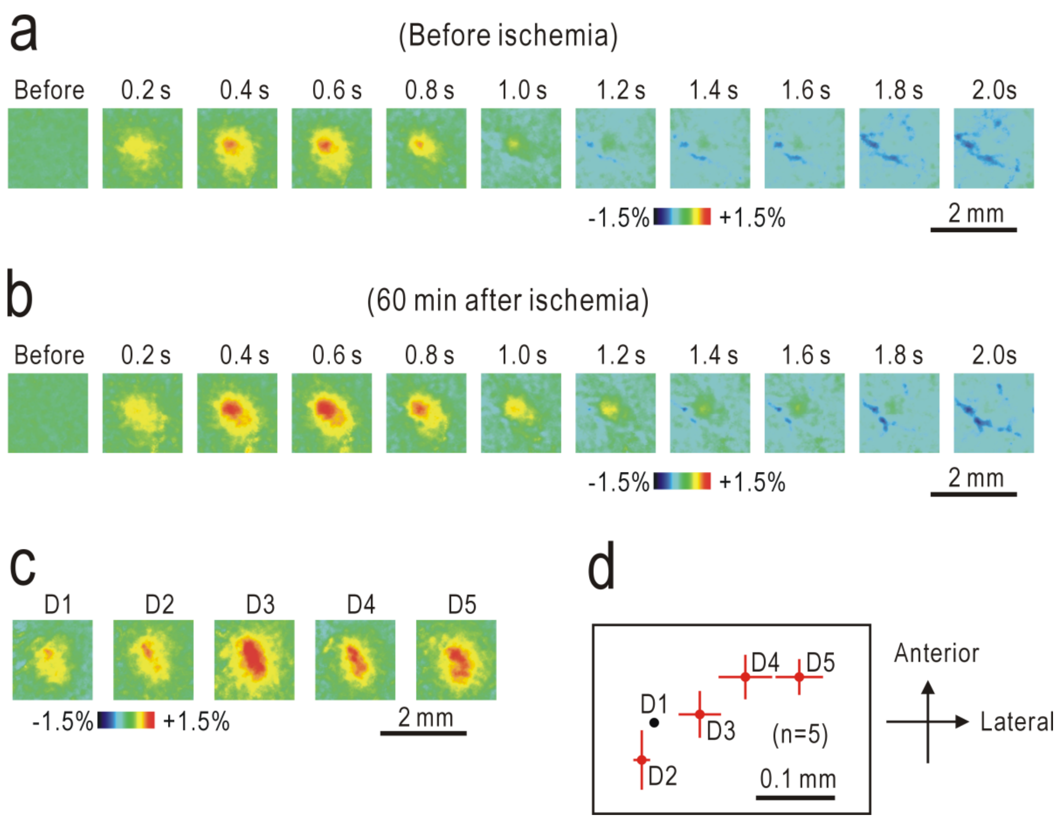

Supplementary Figure S3. Properties of cortical responses. (a) Sequential cortical images shown at 0.2 s intervals before ischemic treatment. **(b)** Sequential images shown at 0.2 s intervals 60 min after ischemic treatment. These images were obtained from the same mouse shown in Fig. 3b. **(c)** Example of contralateral cortical responses elicited by each toe of the left hindpaw. **(d)** Somatotopic maps obtained from five mice. The locations of the response center relative to that of the responses elicited by stimulation applied to the first toe (D1) were plotted. Mean ± SEM are shown.

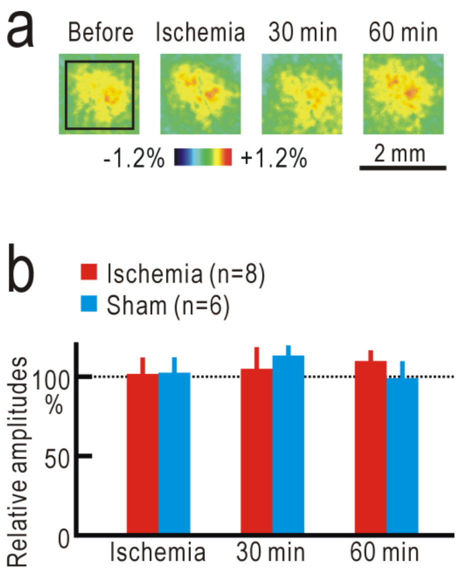

Supplementary Figure S4. Cortical responses to forepaw stimulation contralateral to hindpaw ischemia. (a) Example of S1 responses to stimulation applied to the left forepaw before, during, and after ischemic treatment applied to the left hindpaw. **(b)** Relative amplitudes of the responses to stimulation applied to the left forepaw.
